# Evaluating the Effects of Gamma-Irradiation for Decontamination of Medicinal Cannabis

**DOI:** 10.3389/fphar.2016.00108

**Published:** 2016-04-27

**Authors:** Arno Hazekamp

**Affiliations:** Head of Research and Education, Bedrocan International BVVeendam, Netherlands

**Keywords:** medicinal cannabis, cannabinoids, terpenes, gamma-irradiation, quality control

## Abstract

In several countries with a National medicinal cannabis program, pharmaceutical regulations specify that herbal cannabis products must adhere to strict safety standards regarding microbial contamination. Treatment by gamma irradiation currently seems the only method available to meet these requirements. We evaluated the effects of irradiation treatment of four different cannabis varieties covering different chemical compositions. Samples were compared before and after standard gamma-irradiation treatment by performing quantitative UPLC analysis of major cannabinoids, as well as qualitative GC analysis of full cannabinoid and terpene profiles. In addition, water content and microscopic appearance of the cannabis flowers was evaluated. This study found that treatment did not cause changes in the content of THC and CBD, generally considered as the most important therapeutically active components of medicinal cannabis. Likewise, the water content and the microscopic structure of the dried cannabis flowers were not altered by standard irradiation protocol in the cannabis varieties studied. The effect of gamma-irradiation was limited to a reduction of some terpenes present in the cannabis, but keeping the terpene profile qualitatively the same. Based on the results presented in this report, gamma irradiation of herbal cannabis remains the recommended method of decontamination, at least until other more generally accepted methods have been developed and validated.

## Introduction

Because medicinal cannabis is often used by chronically ill patients affected by a weakened immune system, pharmaceutical regulations in countries such as The Netherlands and Canada specify that these products must adhere to strict safety standards regarding microbial contamination. When harmful microbes or fungal spores are inhaled during e.g., vaporizing or smoking, they may directly enter the bloodstream and cause opportunistic infections. Such contamination risks are not merely hypothetical: cases of chronic pulmonary aspergillosis associated with smoking unsafe cannabis are well established in the scientific literature (Llamas et al., 1978; Sutton et al., 1986; Marks et al., 1996; Szyper-Kravitz et al., 2001; Kouevidjin et al., 2003; Cescon et al., 2008; Bal et al., 2010; Ruchlemer et al., 2015). For those with compromised immune systems, such lung diseases could be even fatal (Hamadeh et al., 1988).

To minimize contamination risks to patients, Dutch regulations demand that medicinal cannabis contains no more than 100 colony-forming units (CFUs) per gram of final product, which is close to sterility^1^. Under the Canadian program, limits are somewhat higher with a maximum of 1.000 CFUs per gram^2^. Following European or US Pharmacopoeia standards for inhaled preparations, certain specific pathogens must be completely absent, i.e., *Staphylococcus aureus, Pseudomonas aeruginosa*, and any bile-tolerant Gram-negative bacteria such as *E. coli* (EP, 2015; USP, 2015). Furthermore, the absence of fungal mycotoxins must be confirmed by additional quality control testing.

Decontamination of medicinal (herbal) cannabis is a necessity, as it has yet not been possible to grow cannabis plants under sufficiently sterile conditions to keep contamination levels below the required safety limits. Even if this were feasible, the multiple steps involved in harvesting, drying, processing and packaging cannabis buds would make it extremely hard to maintain near-sterile conditions throughout the entire production procedure. As a result, medicinal cannabis in The Netherlands as well as in Canada is treated by gamma irradiation before it becomes available to patients^1, 2^.

### Methods of decontamination

Reduction of microbes can be achieved by various treatments, as listed in Table 1. The optimal choice of decontamination depends on the nature of the product to be treated. For herbal materials such as cannabis, the only currently viable option for treatment is the use of ionizing radiation. Any of the other decontamination treatments would either affect chemical content or texture (i.e., heat, chemicals, pressure, steam; Ruchlemer et al., 2015) or would not penetrate beyond the surface of the dense cannabis flowers (i.e., UV-light).

**Table 1:** **List of current main methods available for decontamination or sterilization of (food) products**.

**Type of decontamination**	**Main treatments**
Heat:	Dry heat
	Steam (autoclave)
Chemicals:	Gas (ethylene oxide, ozone, nitrogen dioxide)
	Liquid (hydrogen peroxide, formaldehyde)
High pressure:	Pascalization
Filtration:	Micropore filter (*NB*: for liquids only)
Radiation:	Non-ionizing (UV-light)
	Ionizing (gamma-irradiation, X-rays, electron beam)

Gamma irradiation involves exposing the target material to packets of light (photons) that are so highly energetic (gamma rays) that they damage the DNA strands present in microbes. As a result, the affected microbes cannot multiply, and consequently they will perish^3^. Because medicinal cannabis is a harvested and dried (i.e., non-living) product, this effect is not relevant for the condition of the cannabis plant cells.

### Irradiation safety and concerns

Most commonly, the radioactive element cobalt-60 (^60^Co) is used as the source for gamma irradiation. If administered at appropriate levels, irradiation can be used for the removal of decay-causing bacteria from many foods and herbs, and can prevent sprouting of fruit and vegetables to maintain freshness and flavor (EFSA Panel on Food Contact Materials Enzymes Flavourings Processing Aids-CEF, 2011; Arvanitoyannis et al., 2009). Decontamination or sterilization by gamma irradiation is also widely applied to medical instruments and medicines (Hasanain et al., 2014).

Over the years, the safety of irradiated foods has been confirmed in various animal as well as human studies. These include animal feeding studies lasting for several generations in several different species, including mice, rats and dogs (WHO, 1999; EFSA Panel on Food Contact Materials Enzymes Flavourings Processing Aids-CEF, 2011). NASA astronauts have been eating irradiated foods when they fly in space since the 1970s (Perchonok and Bourland, 2002). Irradiation-induced changes in food components are generally small and not significantly different from those reported in other conventional preservation processes, especially those based on thermal treatment (EFSA Panel on Food Contact Materials Enzymes Flavourings Processing Aids-CEF, 2011; Shahbaz et al., 2015). The changes in some components that are sensitive to irradiation, like some vitamins or micronutrients (Caulfield et al., 2008) may be minimized by using proper treatment conditions (Kilcast, 1994; WHO, 1999).

The safety of irradiated foods has been endorsed by the World Health Organization (WHO), the Food and Agriculture Organization of the United Nations (FAO), the U.S. Department of Agriculture (USDA), Health Canada (HC), the European Union (EU), and the Food and Drug Administration (FDA). Gamma irradiation is now permitted by over 60 countries with at least 400,000 metric tons of foodstuffs annually processed worldwide (EFSA Panel on Food Contact Materials Enzymes Flavourings Processing Aids-CEF, 2011). The regulations that dictate how food is to be irradiated, as well as which foods are allowed to be treated, may vary greatly from country to country^4^.

Despite these developments, irradiation remains a somewhat controversial decontamination technique that can spark emotional debates among the general public. One specific concern with irradiation treatment is the formation of radiolytic compounds, in particular 2-alkylcyclobutanones (2-ACBs). These chemicals are formed in minute quantities when high fat containing foods (such as sesame seeds, pork meat, cheese, eggs, fish) are subjected to gamma irradiation, and their content increases with irradiation dose (Zanardi et al., 2007; Lee et al., 2008). Although some contradictory *in vitro* findings exist on the safety of these compounds, overall scientific consensus is that 2-ACBs are not an immediate cause for concern (EFSA Panel on Food Contact Materials Enzymes Flavourings Processing Aids-CEF, 2011).

Of course, consumers may also be concerned about the indirect effects of irradiation, such as the way it changes the way we relate to food or herbal medicine, or how the use of radioactive materials affect the environment during their mining, shipping and use. Furthermore, irradiation, like any form of treatment, adds to the final cost of a food product or medicine. All these concerns should be taken into consideration when determining whether gamma irradiation is the proper choice for decontamination of a product.

### Evaluating the effects of gamma irradiation on medicinal cannabis

Patients have occasionally expressed their concerns about the effects of irradiation treatment on medicinal cannabis. Some have claimed a change of taste or effect, while others worry about changes in the chemical composition or the quality of their medicine^5^. In response to such concerns, some Canadian licensed producers of medicinal cannabis initially pledged not to apply irradiation, but were forced to reconsider when their products could not meet microbial safety requirements. To cushion the impact on their customers, the obscuring term “cold pasteurization” was introduced when in fact gamma irradiation treatment was applied^6^.

In fresh Cilantro leaves, gamma irradiation was shown to reduce the content of terpenes such as myrcene and linalool (Fan and Sokorai, 2002). Likewise, irradiation may perhaps have an effect on cannabis terpenes, which seem to play an important role in the synergistic effect and bioavailability of cannabinoids (Russo, 2011). Although an early study by our group on the effect of cannabis irradiation did not indicate changes in the cannabinoid profile (unpublished data), chromatographic analysis of cannabinoids has significantly improved over the years meaning that more detailed changes in the cannabinoid profile may now be visualized. The occurrence of 2-ACBs seems of limited relevance in the case of cannabis, because average daily cannabis consumption is very small compared to other irradiated products such as meats, fruits of vegetables. Also, cannabis flowers do not contain significant amounts of fat needed to form these radiolytic compounds in the first place.

To address the concerns that may exist around gamma irradiation of medicinal cannabis, we evaluated the effects of irradiation treatment of four different cannabis varieties covering different compositions (THC vs. CBD dominant types, Sativa vs. Indica types). Samples were compared before and right after standard gamma-irradiation treatment, by performing quantitative analysis of major cannabinoids, as well as qualitative analysis of full cannabinoid and terpene profiles. In addition, water content and microscopic appearance of the cannabis flowers was evaluated.

## Materials and methods

### Solvents and chemicals

All organic solvents were HPLC or analytical grade. Acetonitrile was obtained from Boom labs BV (Meppel, The Netherlands). Ethanol and phosphorus pentoxide (P_4_O_10_) was purchased from VWR (Amsterdam, The Netherlands).

### Cannabis samples

Pharmaceutical-grade cannabis was obtained from the licensed Dutch cultivator, Bedrocan BV (Veendam, the Netherlands). Plants were grown from genetically identical clones under standardized indoor conditions. Flower tops were harvested and air-dried for 1 week under controlled temperature and humidity. Four different standardized varieties available in Dutch pharmacies were used for this study i.e., *Bedrocan*®, *Bediol*®, *Bedica*®, and *Bedrolite*®. Batch information and chemical composition of these products is listed in Table 2.

**Table 2:** **Cannabis type and batch information of the cannabis varieties used in this study**.

**Variety name**	**Batch #**	**THC/CBD type**	**Sativa/Indica type**	**Harvest date**
*Bedrocan*	A1.01.45	THC	Sativa	11-12-2014
*Bediol*	A2.05.15	THC + CBD	Sativa	25-12-2014
*Bedrolite*	A2.08.13	CBD	Sativa	08-01-2015
*Bedica*	A2.07.20	THC	Indica	22-01-2015

All cannabis batches used for this study were harvested in the period of late 2014–early 2015. Following standard procedure, each batch was packaged in portions of 250 grams in triple laminate foil bags with zip-lock closure (type Lamizip aluminum; Daklapack, The Netherlands) for gamma irradiation treatment at Synergy Health (Etten-Leur, The Netherlands). Each batch received an irradiation dose of (minimum) 10 kGy produced with a Cobalt-60 radiation source.

Of each cannabis variety a 10 gram sample was collected before (non-irradiated control) as well as after (irradiated sample) gamma irradiation, resulting in a total of 8 samples for this study [4 varieties × 2 treatments (before/after irradiation)]. Samples were homogenized by grinding in a blender until the material was about 5 mm in diameter. Ground samples were finally used for determination of water content, and for sample extraction for GC/UPLC analysis. Of variety *Bedrocan*, the most popular variety used by Dutch patients (Hazekamp and Heerdink, 2013), some non-homogenized samples were kept for microscopic analysis.

All samples were handled and stored under equivalent conditions. For each variety, irradiated and control samples were extracted and analyzed on the same day, so that any changes in chemical composition could only be attributed to the irradiation treatment. This study was carried out under a cannabis research license issued by the Dutch Health Department.

### Water content determination

Water content of each homogenized sample was determined by using the Loss on Drying (LOD) method according to EP monograph 2.3.32 (method C). In short, 500 mg of each sample (in duplicate) was accurately weighed in small plastic containers, and dried for 24 h at 40°C under vacuum inside a desiccator containing the potent desiccant phosphorus pentoxide. Subsequently, all samples were weighed again. Water content (in percentage of initial weight) was determined by comparing weight before and after the procedure.

### Sample extraction

Ground cannabis samples were extracted for Gas Chromatography (GC) and Ultra-Performance Liquid Chromatography (UPLC) analysis as described in the Dutch Analvtical Monograph for release testing of Cannabis Flos, version 7.1 (OMC, 2015)^7^. In short, 1000 mg of each homogenized sample (in duplicate) was extracted with 40 mL of absolute ethanol in plastic serum tubes (maximum content 50 mL) while mechanically shaking for 15 min at 300 rpm. Tubes were then centrifuged at 3000 rpm and clear supernatant was transferred to a 100 mL volumetric flask. For exhaustive extraction, the procedure was repeated twice more with 25 mL of ethanol, and supernatants were combined. Volumes were adjusted to 100 mL with ethanol, mixed well, and filtered through a 0.45 μm PTFE syringe filter to remove small particles. Filtrated extracts were used directly for GC analysis, or further diluted with acetonitrile/water (70:30, v/v) for analysis by UPLC.

### Quantitative UPLC analysis of major cannabinoids

The UPLC profiles were acquired on a Waters (Milford, MA) Acquity UPLC system consisting of a gradient pump, an autosampler, a column oven and a diode array detector (DAD). The device was controlled by Waters Empower software. Full spectra were recorded in the range of 200–400 nm. The analytical column was a Waters Aquity C_18_ (1.7 μm, 2.1 × 150 mm) equipped with a matching guard column. The mobile phase consisted of a gradient of acetonitrile (A) and water (B), both containing 0.1% formic acid. The gradient was programmed as follows: 0–6 min (hold at 70% A); 6–10.5 min (linear increase to 100% A); 10.5–11 min (hold at 100% A). The column was then re-equilibrated under initial conditions for 1.5 min, resulting in a total runtime was 12.5 min. Flow-rate was 0.4 mL/min. Injection volume was 10 μL. Chromatographic peaks were recorded at 228 nm. All determinations were carried out at 30°C. All samples were analyzed in duplicate.

**Figure 1:**
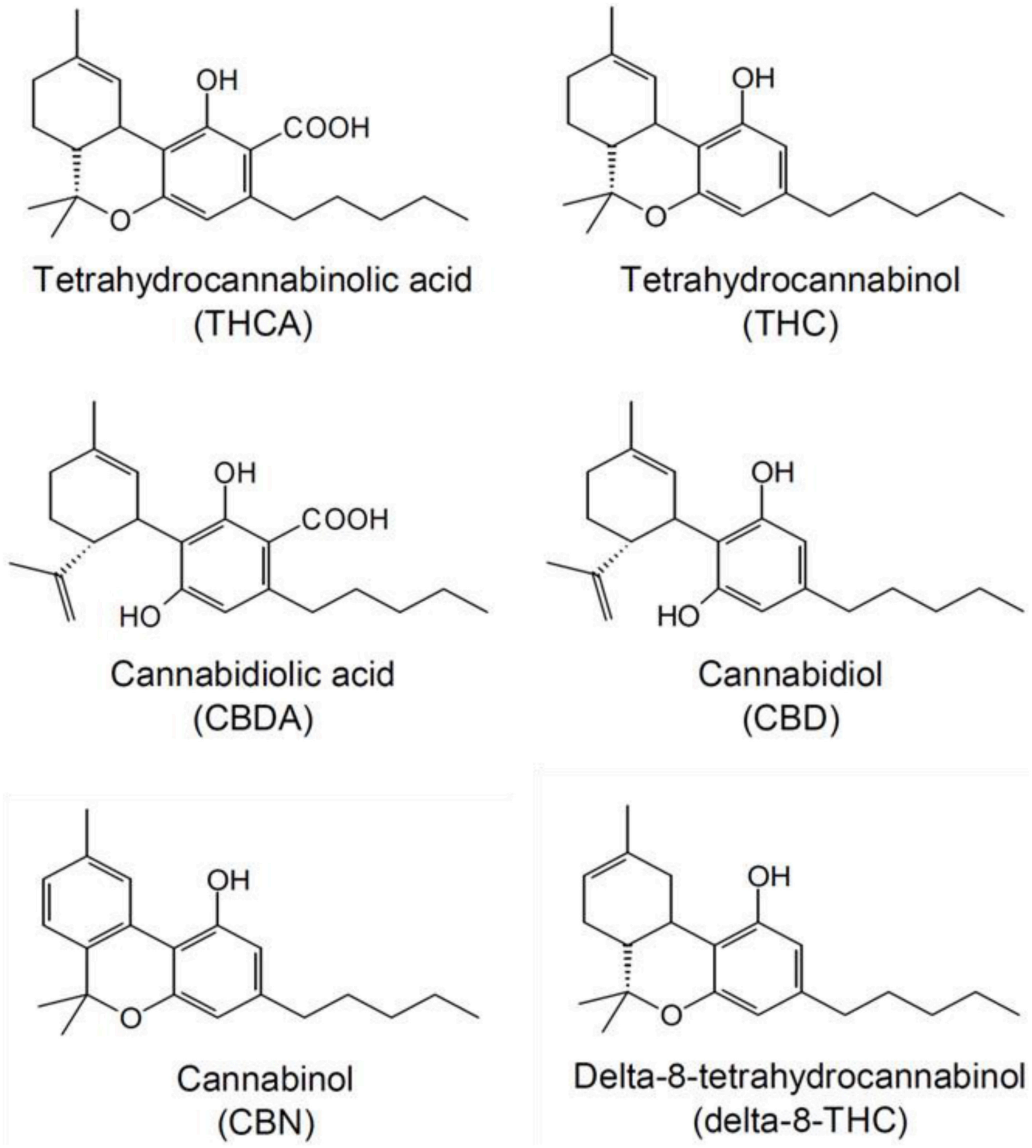
**Structures of the cannabinoids quantitatively analyzed by UPLC**.

Applying the standard protocol for release testing of medicinal cannabis (OMC, 2015)^7^, the following cannabinoids were quantitatively determined: THC, THCA, CBD, CBDA, delta-8-THC, CBN. The structures of these compounds, including their full chemical names, are shown in Figure 1.

### Qualitative GC analysis of cannabinoid and terpene profiles

Gas chromatography was used for the simultaneous qualitative analysis of monoterpenes, sesquiterpenes, and cannabinoids as previously reported (Hazekamp and Fischedick, 2012). An Agilent GC 6890 series (Agilent Technologies Inc., Santa Clara, CA, USA) equipped with a 7683 autosampler and a flame ionization detector (FID) was used. The instrument was equipped with a DB5 capillary column (30 m length, 0.25 mm internal diameter, film thickness 0.25 μm; J&W Scientific Inc., Folsom, CA, USA). The injector temperature was 230°C, with an injection volume of 1 μl, a split ratio of 1:20 and a carrier gas (N_2_) flow rate of 1.2 ml/min. The temperature gradient started at 60°C and linearly increased at a rate of 3°C/min until the final temperature of 240°C which was held for 5 min resulting in a total run time of 65 min/sample. The FID detector temperature was set to 250°C. The device was controlled by Agilent GC Chemstation software version B.04.01.

### Microscopic visualization of glandular hairs

In order to visualize potential morphological changes in the glandular hairs (where cannabinoids and terpenes are produced) present in the cannabis flowers, microscopic analysis of cannabis variety *Bedrocan* was performed before and after gamma-irradiation treatment. Whole cannabis flowers were used, without homogenizing. A Leica (type MZ16FA) stereo-microscope was used. Images were captured at a magnification factor ranging from 20 to 120 times with a Leica (type DFC420C) camera, controlled by LAS software.

## Results

### Loss on drying

**Figure 2:**
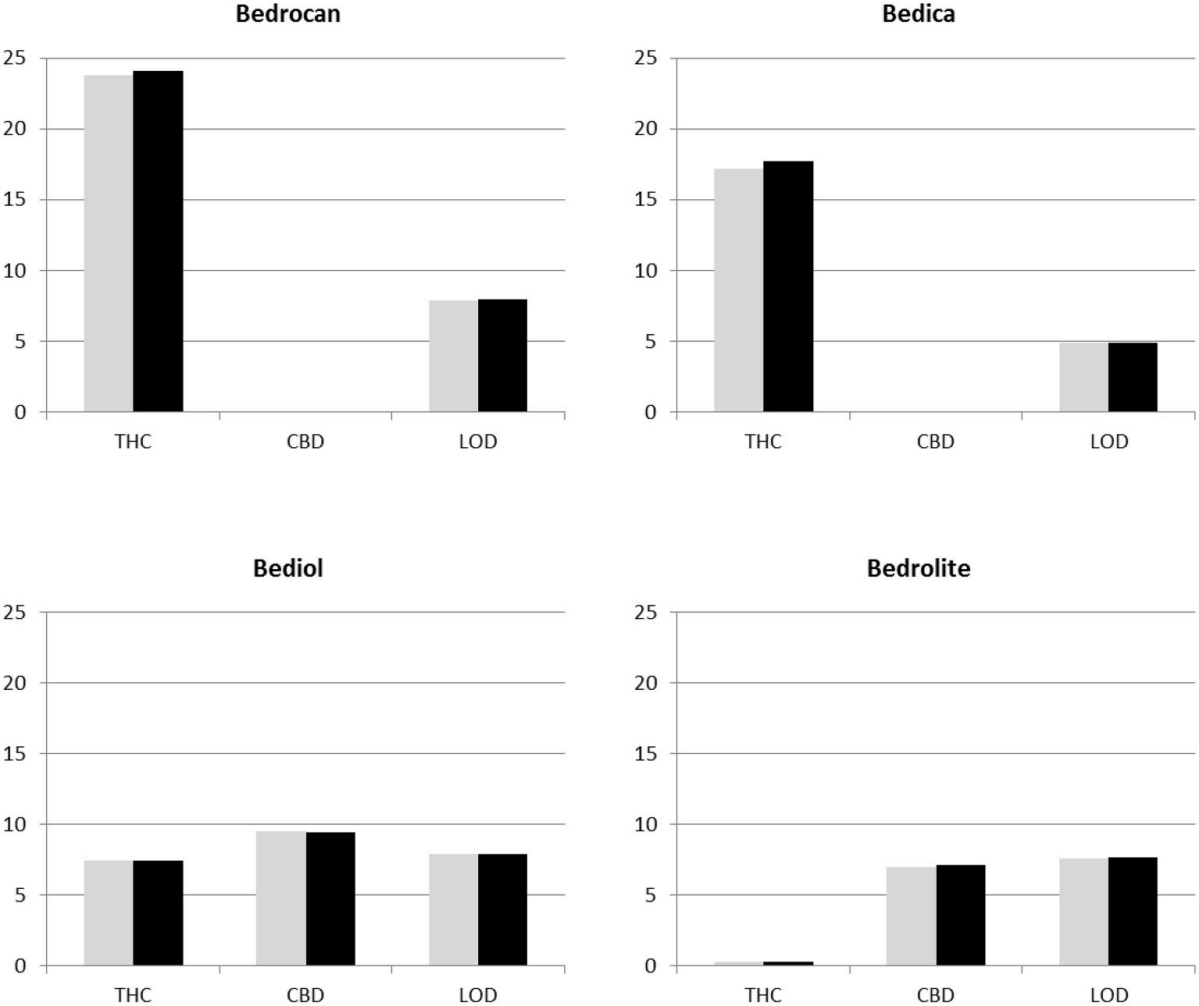
**Total THC and total CBD content (in % of dry weight) as determined by UPLC analysis, as well as water content (in % of total weight) as determined by Loss on Drying method (LOD) in all studied varieties ***before*** (gray bars) and ***after*** (black bars) irradiation treatment**.

Inhalation, either by smoking or vaporizing, is currently the main mode of administration used by patients (Hazekamp et al., 2013). Water content (humidity) seems to have significant impact on how consumers appreciate medicinal cannabis products during inhalation (Ware et al., 2006). Although gamma irradiation does not significantly heat up the treated product, water may be lost during the procedure either as a result of the irradiation itself (Yu and Wang, 2007) or because of shipping and handling of the product during the treatment. Release specifications for Bedrocan products require the water content to be no more than 10%. As shown in Figure 2, the actual water content of the analyzed varieties ranged between 5 and 8%, with no differences between treated and control samples.

### UPLC analysis

Six major cannabinoids were quantitatively analyzed by applying a validated UPLC methodology that is used as standard procedure for release testing of medicinal cannabis in The Netherlands. As customary, the sum of THC and its acidic precursor THCA is reported as “total THC content.” Similarly, the sum of CBD and CBDA is reported as “total CBD content.” It should be noted that delta-8-THC and CBN are not originally produced by the cannabis plant, but are formed as degradation products of THC by exposure to heat or light, or by prolonged storage (Hazekamp et al., 2010).

Results of cannabinoid testing are shown in Figure 2, indicating that levels of total THC and/or CBD were not altered by irradiation treatment in any of the varieties studied. No delta-8-THC or CBN was detected in any of the samples (before or after irradiation) at levels over 0.1% (which equals 1 mg/gram of cannabis flower).

### GC analysis

**Figure 3:**
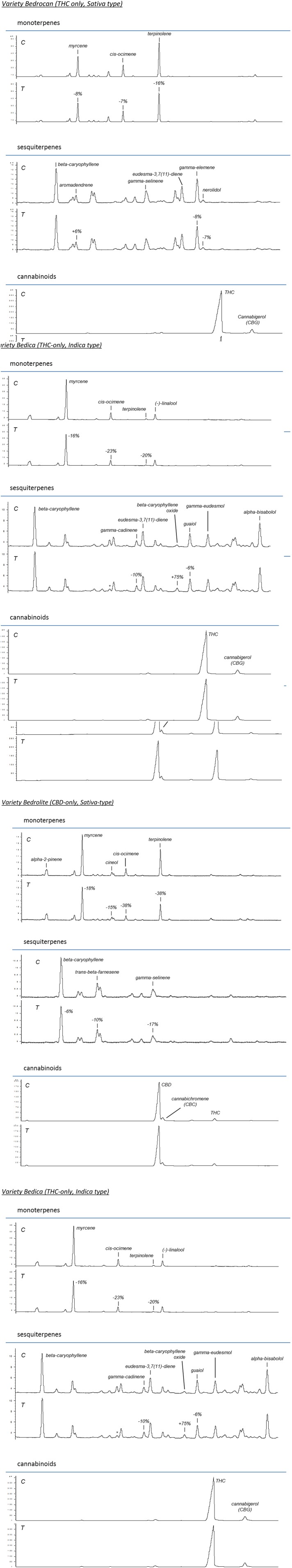
**GC profiles of four studied varieties showing monoterpenes, sesquiterpenes, and cannabinoids in separate sections**. C, control (non-irradiated); T, treated (irradiated); ^*^: artifact. Numbers indicate percentage of change in treated samples compared to non-treated controls.

Components visualized by GC analysis were not individually quantified because of the multitude of chromatographic peaks of interest (>50). Instead, the entire profiles of all visible peaks are presented in Figure 3. Because of the complexity of these profiles, the sections of the profile where monoterpenes, sesquiterpenes, and cannabinoids elute are displayed separately. For each variety, control (non-irradiated) samples, and treated (irradiated) samples are shown side by side, using the same vertical scale to allow direct comparison. The main peaks in each variety were identified based on previously published data (Hazekamp and Fischedick, 2012).

While the overall qualitative composition of the samples was unaltered, differences in several terpene components could be detected after irradiation in the cannabis varieties studied. Components that showed a clear reduction after irradiation treatment are indicated in Figure 3 by showing the relative change (in %) compared to untreated sample. Because a small variability of terpene content between samples is to be expected, and is also observed between replicates of non-treated samples, changes that are smaller than +/– 5% are not indicated. The main components affected were the monoterpenes myrcene, cis-ocimene and terpinolene, and the sesquiterpenes gamma-selinene, eudesma-3,7(11)-diene and gamma-selinene. No new terpene peaks were formed as a result of treatment. No cannabinoids were altered or formed as a result of irradiation.

### Microscopy

**Figure 4:**
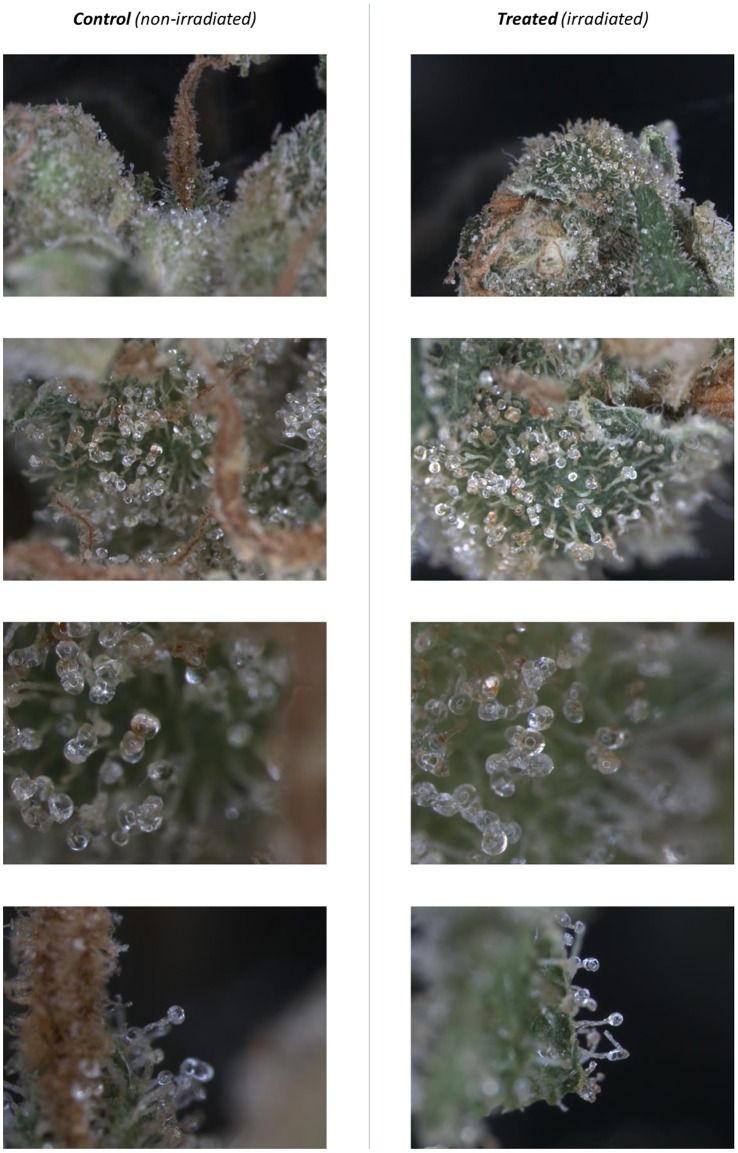
**Microscopic images of trichomes (glandular hairs) before and after treatment with gamma-irradiation**. Cannabis variety *Bedrocan* was used. Magnification ± 20–120 times.

Multiple microscopic images were obtained of variety *Bedrocan* on flowers collected before and after treatment with gamma-irradiation, at a magnification of about 20–120 times. The trichomes (glandular hairs) where cannabinoid and terpenes are excreted by the cannabis plant are clearly visible, as shown in Figure 4. No clear differences in trichome structure, color, density, or shape could be observed between the control (non-irradiated) samples and treated (irradiated) samples.

## Discussion and conclusion

Gamma irradiation treatment of cannabis has become standard practice in the government-supported medicinal cannabis programs of The Netherlands as well as Canada. In the study presented here such treatment, at a radiation dose (10 kGy) sufficient to reduce microbial contamination (bioburden) to pharmaceutically acceptable levels, did not cause any changes in the content of THC and CBD, generally considered as the most important therapeutically active components of medicinal cannabis (Grotenhermen and Müller-Vahl, 2012). Likewise, the water content and the microscopic structure of the dried cannabis flowers were not altered by standard irradiation protocol in four different cannabis varieties. The study included representative varieties of THC and CBD dominant types, as well as Sativa and Indica types.

In our study, irradiation had a measurable effect on the content of multiple cannabis terpenes, mainly on the more volatile monoterpenes. Reduction of affected terpenes was in general between 10 and 20%, but for some components this may be as much as 38%. In a previous study evaluating the effect of gamma irradiation on fresh Cilantro, a decrease in terpene content was also described (Fan and Sokorai, 2002). However, the authors concluded that the observed loss of terpenes such as myrcene and linalool was insignificant compared to the losses that occurred by evaporation during refrigerated storage of Cilantro. Also in orange juice the effect of irradiation on terpenes was found to be non-significant in comparison to changes induced by refrigerated storage (Fan and Gates, 2001). Likewise, the slight terpene reduction observed in the current study is comparable to the effect that short term storage in a paper bag had on cannabis samples, in a study performed by (Ross and ElSohly, 1996). A likely explanation therefore seems that gamma irradiation slightly accelerates the evaporation of some of the more volatile terpenes. This idea is supported by the fact that no degradation products or additional chromatographic peaks were found to account for the lost terpenes, with the exception of some beta-caryophyllene oxide formed in the irradiated sample of variety *Bedica*. Interestingly, terpenes were not affected to the same degree in all varieties, e.g., myrcene content was clearly reduced in varieties *Bedica* and *Bedrolite* but not in variety *Bediol*. Perhaps this indicates a protective effect that cannabis components may have on each other when present in specific proportions.

Some cannabis users have claimed that irradiation changes the taste and/or smell of cannabis during smoking or vaporizing (personal observation by the author). Unfortunately, such opinions may be hard to substantiate because the same cannabis is usually not available to consumers in both its irradiated and non-irradiated form to allow direct comparison, meaning there is no “base-line” product to quantify the magnitude of the change. Nevertheless, the taste and smell of cannabis mainly depends on its terpene (essential oil) content (Russo, 2011). While the current study indicated quantitative changes in some of the terpenes upon irradiation, a subtle change in smell or taste may indeed be possible as a result of such treatment. Despite these changes, the overall terpene profile of each variety remained clearly recognizable.

Gamma irradiation remains controversial among some consumers of medicinal cannabis. However, weighing the risks vs. the benefits currently keeps pointing toward the use of this decontamination procedure. After all, cannabis plants cannot (yet) be grown and processed under conditions aseptic enough to meet pharmaceutical standards, while infection risks are well documented in the medical literature and can be harmful or even fatal to seriously ill patients. Meanwhile, the main harm of gamma-irradiation seems to be limited to a reduction of some terpenes present in the cannabis, leading to a small quantitative effect, but keeping the terpene profile qualitatively essentially intact.

Based on the results presented in this report, gamma irradiation of herbal cannabis remains the recommended method of decontamination, at least until other more generally accepted methods have been developed and validated. This is especially important when cannabis is prescribed to seriously ill and possibly immune-deprived patients, with an increased risk of suffering from microbial infection. Meanwhile, the development of improved hygienic standards for cultivation and processing of medicinal cannabis may ensure that irradiation doses can be reduced to an absolute minimum. In time, gamma-irradiation may eventually be replaced with other, more generally accepted, forms of reliable decontamination.

## Author contributions

The author confirms being the sole contributor of this work and approved it for publication.

### Conflict of interest statement

The author is full time employed by Bedrocan BV, the licensed company that provided the medicinal grade cannabis used for this study.

## Footnotes

^1^https://www.cannabisbureau.nl/english/specication-sheets

^2^http://www.hc-sc.gc.ca/dhp-mps/marihuana/info/techni-eng.php

^3^http://www.cdc.gov/nczved/divisions/dfbmd/diseases/irradiation_food/#affect_foods

^4^https://nucleus.iaea.org/ifa/

^5^http://www.hc-sc.gc.ca/fn-an/securit/irridation/cyclobutanone-eng.php

^6^http://www.leafscience.com/2014/05/01/tweeds-first-marijuana-orders-delayed-irradiated/

^7^https://www.cannabisbureau.nl/medicinale-cannabis-artsen-en-apothekers-specificaties-en-analysevoorschriften
